# Resveratrol Butyrate Esters Inhibit Obesity Caused by Perinatal Exposure to Bisphenol A in Female Offspring Rats

**DOI:** 10.3390/molecules26134010

**Published:** 2021-06-30

**Authors:** Ming-Kuei Shih, You-Lin Tain, Yu-Wei Chen, Wei-Hsuan Hsu, Yao-Tsung Yeh, Sam K. C. Chang, Jin-Xian Liao, Chih-Yao Hou

**Affiliations:** 1 Graduate Institute of Food Culture and Innovation, National Kaohsiung University of Hospitality and Tourism, No.1, Songhe Rd., Xiaogang Dist., Kaohsiung City 812, Taiwan; mkshih@mail.nkuht.edu.tw; 2 Department of Pediatrics, Kaohsiung Chang Gung Memorial Hospital and Chang Gung University College of Medicine, Kaohsiung 833, Taiwan; tainyl@hotmail.com; 3 Institute for Translational Research in Biomedicine, Kaohsiung Chang Gung Memorial Hospital and Chang Gung University College of Medicine, Kaohsiung 833, Taiwan; 4 Department of Medicine, Chang Gung University, Linkow 333, Taiwan; naosa720928@gmail.com; 5 Department of Food Safety/Hygiene and Risk Management, College of Medicine, National Cheng Kung University, Tainan 704, Taiwan; whhsu@mail.ncku.edu.tw; 6 Aging and Disease Prevention Research Center, Fooyin University, Kaohsiung 831, Taiwan; glycosamine@yahoo.com.tw; 7 Biomed Analysis Center, Fooyin University Hospital, Pingtung 928, Taiwan; 8 Experimental Seafood Processing Laboratory, Costal Research and Extension Center, Mississippi State University, Starkville, MS 39567, USA; schang@fsnhp.msstate.edu; 9 Department of Food Science, Nutrition and Health Promotion, Mississippi State University, Starkville, MS 39762, USA; 10 Department of Seafood Science, National Kaohsiung University of Science and Technology, Kaohsiung 824, Taiwan; j0920181@gmail.com

**Keywords:** resveratrol butyrate esters (RBEs), perinatal exposure, obesity, bisphenol A (BPA), Firmicutes/Bacteroidetes (F/B) ratio

## Abstract

Resveratrol butyrate esters (RBE) are derivatives of resveratrol (RSV) and butyric acid and exhibit biological activity similar to that of RSV but with higher bioavailability. The aim of this study was designed as an animal experiment to explore the effects of RBE on the serum biochemistry, and fat deposits in the offspring rats exposed to bisphenol A (BPA), along with the growth and decline of gut microbiota. We constructed an animal model of perinatal Bisphenol A (BPA) exposure to observe the effects of RBE supplementation on obesity, blood lipids, and intestinal microbiota in female offspring rats. Perinatal exposure to BPA led to weight gain, lipid accumulation, high levels of blood lipids, and deterioration of intestinal microbiota in female offspring rats. RBE supplementation reduced the weight gain and lipid accumulation caused by BPA, optimised the levels of blood lipids, significantly reduced the Firmicutes/Bacteroidetes (F/B) ratio, and increased and decreased the abundance of S24-7 and Lactobacillus, respectively. The analysis of faecal short-chain fatty acid (SCFA) levels revealed that BPA exposure increased the faecal concentration of acetate, which could be reduced via RBE supplementation. However, the faecal concentrations of propionate and butyrate were not only significantly lower than that of acetate, but also did not significantly change in response to BPA exposure or RBE supplementation. Hence, RBE can suppress BPA-induced obesity in female offspring rats, and it demonstrates excellent modulatory activity on intestinal microbiota, with potential applications in perinatological research.

## 1. Introduction

Bisphenol A (BPA) exposure is the most prevalent in the environment [1]. Rahman et al. (2021) summarized several confounding factors that may be directly or indirectly related to human BPA exposure and detailed the disparities between scientifically derived safe dosages of BPA and those designated as “safe” by government regulatory agencies [2]. Exposure to BPA during early development has been associated with the prevalence of various cardiometabolic diseases [3] including obesity [4,5], metabolic syndrome [6], type 2 diabetes [5,7], and cardiovascular diseases [5,8]. The toxicokinetic studies of laboratory animals and humans after oral ingestion of BPA have similar results. BPA is rapidly absorbed from the gastrointestinal tract and undergoes first-pass conjugation to BPA-glucuronide (BPA-gluc) and BPA-sulfate (BPA-sulfate), which are biologically inactive metabolites [9,10,11,12]. The studies after oral intake of BPA or high-BPA diet in humans are consistent with animal data, confirming that the internal exposure of unbound BPA is lower after oral exposure, and BPA-gluc is the main metabolite [13,14,15,16,17,18].

Growing evidence suggests that, due to the phenolic structure of BPA, it interacts with estrogen receptors and acts as either agonist or antagonist via estrogen receptor-dependent signaling pathways [19,20,21,22]. Previous studies have demonstrated the developmental origins of the health and disease (DOHaD) hypothesis [23,24], which highlights the links between the periconceptual, foetal, and early infant phases of life and the subsequent development of adult obesity and related metabolic disorders [25,26]. The lipophilicity of BPA facilitates its entry through placental and blood–brain barriers into the foetus, where it triggers oxidative damage and nerve injury in the brain, thereby affecting brain development and causing permanent foetal brain injury [27]. In addition, previous animal studies have demonstrated that maternal and foetal exposure to BPA also damages the immune system and affects the balance of intestinal microbiota in the offspring [28,29]. There is little doubt that poor diet and lack of exercise contribute to the obesity epidemic; recent studies have identified an estrogen endocrine disrupter chemical BPA that may act as an environmental obesogen and either directly or indirectly influence fat accrual [30]. The plausible explanation may involve sex hormones, genomic and non-genomic pathway involving nuclear estrogen receptors, differing developmental pattern and/or epigenetic influence [31,32,33]. Somm et al. [34] reported that exposure to 70 μg/kg/day of BPA during pregnancy can upregulate the expression of PPARγ, C/EBPα, and LPL in abdominal adipocytes of adult female rats. In addition, body weight only increased in females, in which parametrial white adipose tissue (PWAT) weight increased about threefold. Resveratrol (RSV) can prevent BPA-induced metabolic abnormalities in offspring. Darby et al. (2019) showed that RSV can improve the placental blood flow by increasing the bioavailability of NO, can increase the activity of antioxidant enzymes in foetuses and placentas, and can reduce the rates of inflammation and cell death in placentas to ensure adequate foetal nutrition [35]. Therefore, RSV is often employed in the treatment of metabolic abnormalities and oxidative stress attributed to gestational diabetes in offspring.

The pharmacokinetic study reported by Huang et al. (2019) showed that the blood concentration of RSV peaked within 0.5 to 2 h after oral administration, followed by a rapid decline [36], indicating that RSV is unable to induce adequate pharmacological responses in pregnant women; hence, it is not an ideal clinical drug. We reported novel resveratrol butyrate esters (RBEs) and indicated the esterification of RSV with butyrate that contained RSV (~17.1%), RBE monoester (~47.1%), and RBE diester (~35.0%), which also had better hydrogen peroxide scavenging activity than RSV [37] and could effectively inhibit fatty-acid-induced lipid accumulation in HepG2 cells, with effects similar to those of RSV, but achieved at a lower dose level [38]. Butyrate, one of the short-chain fatty acids (SCFAs), can selectively promote the growth of beneficial bacteria that improve the intestinal barrier’s function [39]. As RBE can be catabolised into RSV and butyrate in the body [40], we also attempted to further increase the concentration of butyrate in the intestinal tract to protect the colonic epithelial cells and nervous tissue in the brain.

Numerous studies have confirmed that intestinal microbiota and obesity are closely associated with metabolic abnormalities in hosts [41]. For example, the Firmicutes/Bacteroidetes (F/B) ratio, which is related to obesity, is lower in obese individuals than in normal individuals [42,43,44,45].

To date, there are still no obesity-related perinatological studies of RBE. Although the sex-specific effects of BPA are well documented, including the differential susceptibility of males and females to different doses of BPA [31,32,46,47,48,49], the underlying mechanism remains unclear [47]. Therefore, we performed perinatal BPA exposure studies to observe the effects of RBE supplementation on obesity-related indicators and intestinal microbiota in female rat offspring to better understand the potential applications of RBE.

## 2. Results

### 2.1. Changes in Body Weight, Abdominal Lipid Weight, Plasma Biochemical Markers, and Adipose Tissues in Female Offspring Rats

Table 1 shows that the perinatal exposure to BPA led to obesity in female offspring with 16%, 77%, and 70% increases in their body weight (BW), abdominal lipid weight (LW), and relative lipid weight (RLW) [(BPA/CN) − 1]*100%, respectively. In addition, the perinatal exposure to BPA also affected their blood biochemical and hormonal compositions with 67%, 16%, 30%, and 25% increases and a 49% decrease in their TG, TC, LDL, leptin, and HDL levels, respectively. These negative effects of perinatal exposure to BPA on female offspring rats were reversed via RBE supplementation, which not only prevented obesity and increased blood lipids and relevant hormones in a dose-dependent manner, but also reduced these health parameters to the same levels or to even lower levels than those observed in the CN group. For example, the statistically significant differences between the B+R30 group and the CN group with respect to LW, RLW, TC, and LDL were favourable. In addition, R30 led to an 11% weight gain ((R30/CN) − 1)*100%) and 19% increase in the blood leptin level ((R30/CN) − 1)*100%) in female offspring rats but did not significantly alter their LW and RLW compared to those observed in the CN group. Hence, the body weight gain caused by R30 was not directly associated with increased body fat or leptin concentration.

**Figure 1:**
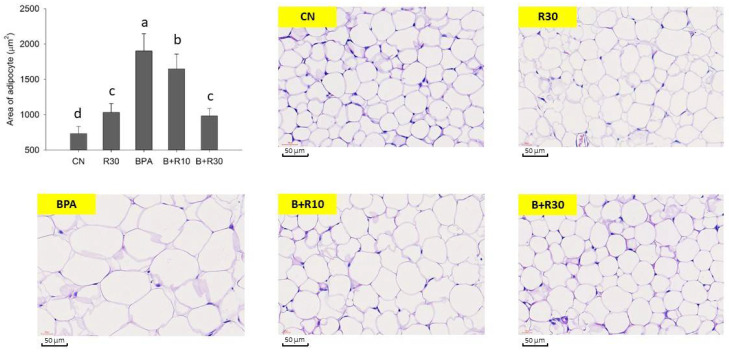
Representative pictures of lipid sections stained with haematoxylin and eosin (H&E) and examined under a microscope. ^a–^^d^ Values with different letters are specific to each group and show significant differences (*p* < 0.05). CN = control group; R30 = group administered with 30 mg/kg/day of RBE; BPA = group administered with 50 μg/kg/day of bisphenol A; BPA+R10 = group administered with 10 mg/kg/day of RBE and bisphenol A; BPA+R30 = group administered with 30 mg/kg/day of RBE and bisphenol A. Data are expressed as mean ± SD (*n* = 4–8).

**Figure 2:**
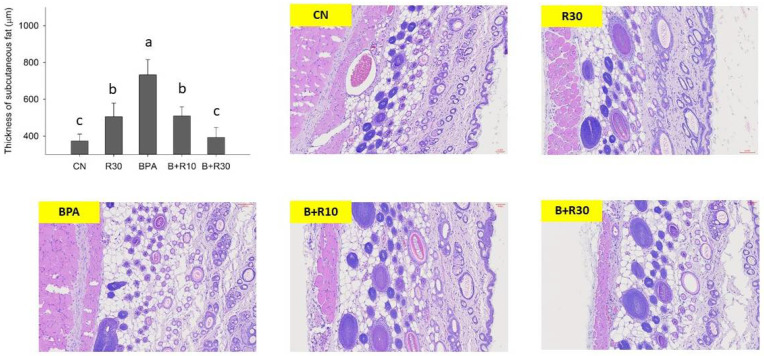
Representative images of subcutaneous tissue sections stained with haematoxylin and eosin (H&E) and examined under a microscope. ^a–^^c^ Values with different letters are specific to each group and show significant differences (*p* < 0.05). CN = control group; R30 = group administered with 30 mg/kg/day of RBE; BPA = group administered 50 μg/kg/day of bisphenol A; BPA+R10 = group administered with 10 mg/kg/day of RBE and bisphenol A; BPA+R30 = group administered with 30 mg/kg/day of RBE and bisphenol A. Data are expressed as means ± SD (*n* = 4–8).

Figure 1 and Figure 2 show the stained sections of white adipose tissue and subcutaneous tissue from the abdomens of female offspring rats. Figure 1 shows that BPA-exposed female offspring rats had significantly abnormally large adipocytes, which were reduced via BRE supplementation (B+R10 and B+R30 groups), indicating that RBE could reduce the BPA-induced adipocyte accumulation in offspring in a dose-dependent manner. In contrast, the staining of the subcutaneous tissue sections of female offspring rats (Figure 2) revealed that, in the BPA/RBE groups, the thickness of the subcutaneous tissue was significantly increased by BPA and could be reduced by RBE. Female offspring rats in the R30 group had slightly thicker subcutaneous tissues than those in the CN group. Figure 1 and Figure 2 generally show similar results. Additionally, the results in Figure 1 and Figure 2 are consistent with the increases in LW and BW demonstrated in Table 1.

Therefore, the above data suggest that obesity in female offspring rats is indeed attributed to BPA and can be significantly suppressed via RBE supplementation. This study, which explored RBE supplementation during the perinatal period, is also the first report on the inhibitory activity of RBE against BPA-induced obesity and its effects on routine blood biochemical markers in offspring.

### 2.2. Changes in Intestinal Microbiota and Faecal Concentrations of SCFAs in Female Offspring Rats

**Figure 3:**
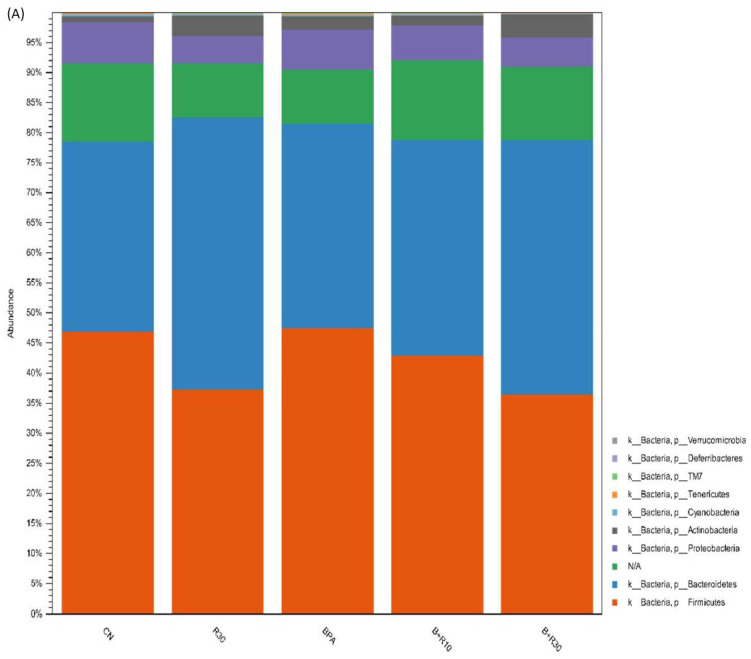

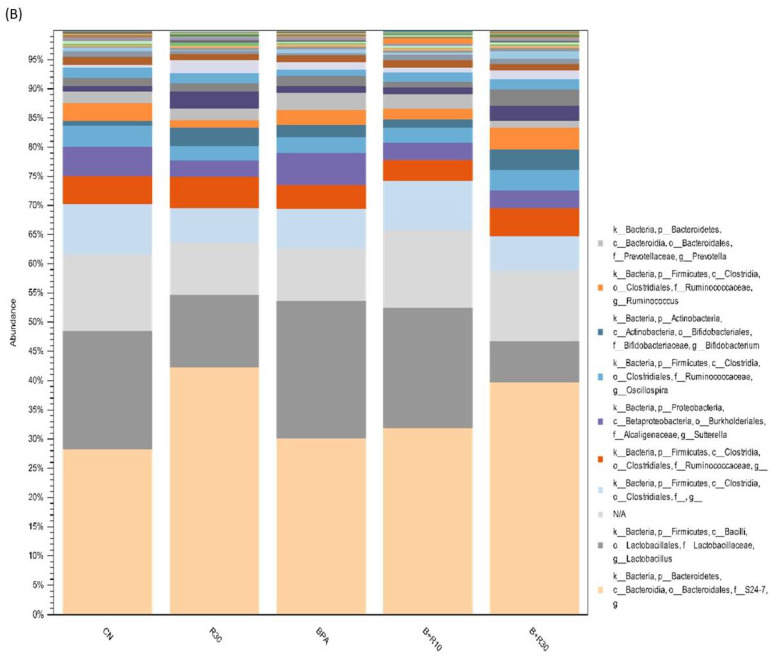

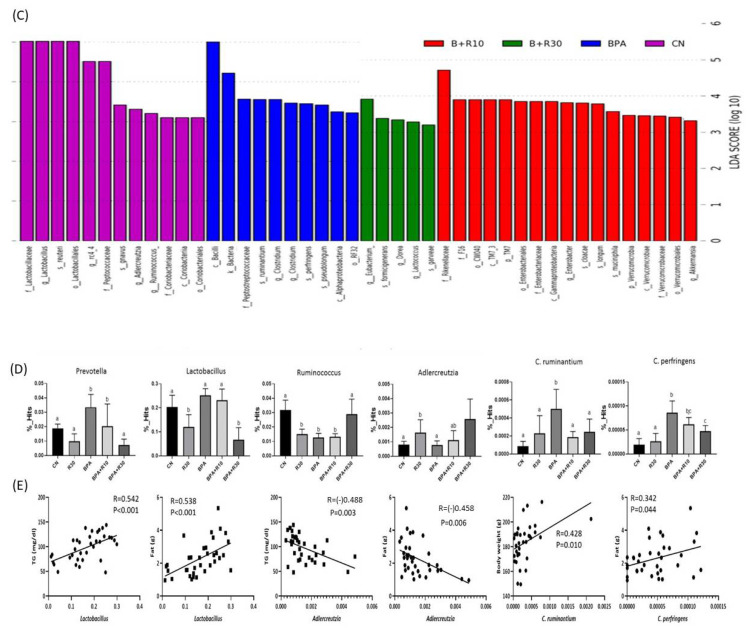
Effects of BPA and RBE on the gut microbiota of female offspring rats. (**A**) The gut bacterial composition at the phylum level. (**B**,**C**) Biomarker taxons generated from the LEfSe analysis (LDA > 3). (**D**) Comparison of the growth and decline of specific strains with significant changes in the OTU and LEfSe analyses. (**E**) Correlation analysis. ^a,^^b^ Values with different letters are specific to each group and show significant differences (*p* < 0.05). CN = control group; R30 = group administered with 30 mg/kg/day of RBE; BPA = group administered with 50 μg/kg/day of bisphenol A; BPA+R10 = group administered with 10 mg/kg/day of RBE and bisphenol A; BPA+R30 = group administered with 30 mg/kg/day of RBE and bisphenol A. Data are expressed as means ± SD (*n* = 4–8).

In this study, we observed changes in the intestinal microbiota and faecal concentrations of SCFAs in female offspring rats. Figure 3A shows that the F/B ratios in CN, BPA, and R30 were 1.45, 1.40, and 0.82, respectively, and those in BPA+R10 and BPA+R30 were 1.21 and 0.86. Therefore, there was a significant dose-dependent increase in the abundance of Bacteroidetes and a decrease in the abundance of Firmicutes after the RBE intake (B+R30 and R30 groups). The results of the operation taxonomic units (OTU) showed that the RBE intake increased the abundance of S24-7 while reducing the abundance of Lactobacillus (Figure 3B,C). We selected several bacteria that displayed significant changes during the OTU and LEfSe analyses to further understand the effects of BPA exposure and RBE supplementation on the succession of intestinal microbiota. Figure 3D shows that BPA exposure increased the abundance of Prevotella, *Clostridium perfringens*, and *Clostridium ruminantiums,* while RBE supplementation reduced the abundance of Lactobacillus and Prevotella and reduced the abundance of *Clostridium perfringens* and *Clostridium ruminantiums* to normal levels. In addition, the B+R30 group showed an increased abundance of Ruminococcus and Adlercreutzia. In contrast, our correlation analysis, which was performed to examine the microbiota and plasma biochemical markers (Figure 3E), revealed that the abundance of Lactobacillus was positively correlated with TG concentration and LW, while the abundance of Adlercreutzia was negatively correlated with TG concentration and LW. In addition, we also found that an elevation of the abundance of *Clostridium perfringens* and *Clostridium ruminantiums* could increase the BW and LW of the hosts. Taken together, our results showed that exposure to BPA dysregulated intestinal microbiota; this effect was alleviated via RBE supplementation, suggesting that RBE could effectively modulate intestinal microbiota.

The quantitative results for the faecal SCFAs in female offspring rats demonstrated that the exposure to BPA increased the faecal concentration of acetate, which could be reduced via RBE supplementation (B+R30) (see Table 2). However, the faecal concentrations of propionate and butyrate were not only obviously lower than that of acetate but also did not significantly differ between BPA-exposed and RBE-supplemented rat offspring. In particular, the R30 group showed a significantly higher faecal concentration of acetate than that of the CN group.

## 3. Discussion

### 3.1. RBE Suppressed BPA-Induced Obesity

Numerous previous studies have shown that low-dose exposure to BPA during pregnancy and at the developmental stage can cause obesity [50,51]. Desai et al. (2018) reported that BPA in pregnant women can enter foetuses through the placenta and blood and can induce the differentiation of hypothalamic neurons in offspring, resulting in a higher number of appetite-promoting AgRP and NPY neurons, which cause obesity in the offspring [52]. BPA exposure also upregulated the expression of adipogenesis factors (e.g., leptin, PPARγ, AP2, and C/EBPα), which accelerated the proliferation and differentiation of adipocytes [53,54]. Here, we found that the RBE intervention improved the BW and LW of female offspring rats with perinatal BPA exposure. As RBE can be hydrolysed by pancreatic lipases into RSV and butyrate in the intestine [40], orally administered RBE not only can demonstrate effects similar to those of RSV but also can prolong the absorption of RSV in the gastrointestinal tract, thereby extending its resident time in the blood. Liu et al. (2020) reported that maternal RSV intervention can prevent the adiposity programmed by maternal and postnatal HFHS diets by inducing metabolic lipid modulation [55]. This study indicates a distinct reprogramming role of the effects of maternal RSV supplements against obesity. RSV treatment prevented leptinaemia and increased p-STAT3 (signal transducer and activator of transcription 3) content in the hypothalamus with no changes in SOCS3 (suppressor of cytokine signalling 3), suggesting an improvement in leptin signalling [56]. Collectively, the data suggest that RSV could reverse hyperleptinemia and improve central leptin action in adult offspring from HF mothers, thus attenuating obesity.

Alway et al. (2017) reported that resveratrol may be useful in enhancing both cellular and functional changes that are common in sarcopenia in order to improve health outcomes in older men and women [57]. RSV supplementation improved muscle mass during reloading after hindlimb suspension and increased the cross-sectional area of type IIA and IIB fibres in response to reloading after hindlimb suspension [58]. Salomão et al. (2019) reported that, in combination with exercise, RSV was beneficial for muscle growth and metabolism, as it increased the expression levels of genes related to muscle anabolism and oxidative metabolism decreased catabolic gene expression [59]. Notably, all of these changes occurred together with muscular hypertrophy and increased body weight. Although RSV can regulate energy metabolism via the AMPK/SIRT1 pathway and is associated with adipogenesis [25], it remains unknown whether RSV can enter the bodies of offspring in trace amounts. Darby et al. (2019) reported that RSV intake during pregnancy enhanced the placental utilization of NO, resulting in vasodilation and improvements in the placental blood flow. This increased the BW of foetuses by enhancing the nutrient and oxygen supply [35]. In other words, the weight gains among female offspring rats compared to the weights observed in the CN group in this study may be associated with improved placental blood flow.

In contrast, RBE potentially prevents blood lipid, cardiovascular, circulatory, and metabolic diseases in offspring. Our results (Table 1) showed that the plasma concentrations of TG, TC, and LDL in female offspring rats increased, while the plasma concentration of HDL decreased after the exposure to BPA. After being supplemented with RBE, female offspring rats in the B+R30 group showed a decline in the plasma concentration of TG and LDL, as well as an increase in the production of HDL. HDL is indispensable for the transport of cholesterol. Upon contacting the surface of HDL particles, the free cholesterol in the blood is rapidly esterified by lecithin–cholesterol acyltransferase (LCAT) and encapsulated in the core of HDL particles for removal from blood circulation [60]. However, LDL, which is considered a risk factor for atherosclerosis, can elevate the blood concentrations of TG and TC. In addition, oxidized LDL can accumulate in blood vessels and exacerbate atherosclerosis [61]. Gingrich et al. (2020) reported that a healthy placenta is crucial for foetal development, and placental abnormalities may easily lead to the onset of cardiovascular diseases and metabolic abnormalities in the foetus [62]. We speculated that exposure to BPA increases the risk for cardiovascular disease in offspring, which may be associated with damages to the placenta caused by the perinatal BPA exposure [62]. As mentioned earlier, RSV can improve the antioxidant defence and NO utilization in the placenta, thereby alleviating the oxidative damage caused by pregnancy complications, such as gestational diabetes, maternal obesity, placental abnormalities, and pre-eclampsia [35]. Based on our results, we infer that perinatal supplementation with RBE signifies the involvement of RSV in the alleviation of damages caused by BPA. Compared with the bioavailability of RSV in the body, RBE can significantly suppress associated injuries at a lower dose [38].

In addition, the expression of leptin (Table 1) in female offspring rats was upregulated after exposure to BPA. The expression of leptin returned to the normal level (similar to the CN group) after supplementation with RBE (B+R10 and B+R30 groups) (*p* > 0.05). However, the R30 group showed a significantly higher level of leptin than the CN group (*p* < 0.05). Administration with RSV alone can increase the concentration of leptin in the body and reduce the elevation of the leptin level and the onset of leptin resistance, which are attributed to obesity or a high-cholesterol diet [63]. Leptin is secreted by adipocytes. Rönn et al. [64] showed that BPA exposure is positively correlated with the serum concentrations of leptin and adiponectin, but has no association with LW, indicating that BPA may interfere with the endocrine function of adipocytes and cause hormonal imbalances. Therefore, we speculated that supplementation with RBE can suppress BPA-induced abnormalities.

Previous studies have shown that PPARγ regulation can be mediated via the ERK1/2 pathway and phosphorylation of PPARγ is involved in insulin sensitivity [65]. Somm et al. [34] reported that exposure to BPA during pregnancy can result in an increase in parametrial white adipose tissue (PWAT) weight in female offspring rats. The expression of PPARγ, C/EBPα, and LPL was upregulated in abdominal adipocytes of adult female rats. Masuno et al. (2001) reported that BPA can increase the activity of LPL and the accumulation of TG in 3T3-L1 cells, leading to its differentiation into adipocytes with larger lipid droplets [66]. The staining of tissue sections in this study also yielded similar results (Figure 1 and Figure 2). BPA increased the size of adipocytes and the thickness of subcutaneous fat tissue, while RBE supplementation (B+R10, B+R30, and R30 groups) significantly reversed the effects of BPA (*p* < 0.05). Li et al. (2017) [67] reported that SIRT1, phospho-ERK1/2, and phospho-PPARγ, adiponectin and BDNF were all dysregulated in rats placed in HH group (maternal high-fat diet + postnatal high-fat diet); administration of resveratrol restored the expression and regulation of these molecules. Their results suggest that maternal high-fat diet during pregnancy and/or lactation sensitizes the offspring to the adverse effects of a subsequent high-fat diet on hippocampal function; however, administration of resveratrol is demonstrated to be beneficial in rescuing these effects [67]. Although there was a statistical difference in R30 compared with the control group, the data showed only a slight difference. We surmised that the R30 group could be related to the offspring’s hemoperfusion [35] during the perinatal period, resulting in better activity and higher food intake. However, the specifics of the possible reasons still need to be further studied and discussed. Although RBE has not yet been extensively studied and its metabolic mechanism in the body remains unknown, our data confirmed that RBE can attenuate obesity caused by BPA exposure in offspring. Such a phenomenon may be attributed to the interference of RBE with the BPA-induced differentiation of adipocytes or its direct effects on the metabolism of adipocytes.

### 3.2. The Relationship between the Suppression of Obesity by RBE and Intestinal Microbiota

Here, the rats were subjected to perinatal BPA exposure to observe the effects of RBE supplementation on obesity-related indicators and intestinal microbiota in their female offspring in order to better understand the potential application of RBE in the future. Accumulating evidence has demonstrated that intestinal microbiota and their metabolites are closely associated with the onset of obesity and metabolic abnormalities in hosts [68]. Intestinal microbiota regulate the energy metabolism of the host and secrete metabolites to affect the health of the host. Therefore, diet-mediated modulation of intestinal microbiota is an important and feasible approach for the improvement of health and disease prevention. Our study revealed that (Figure 3A) the abundance of Bacteroidetes increased significantly, while the abundance of Firmicutes showed a decreasing trend after RBE supplementation. In contrast, Figure 3B,C shows that RBE intake can increase the abundance of S24-7 and decrease the abundance of Lactobacillus.

Li et al. (2021) reported that the Firmicutes/Bacteroidetes (F/B) ratio in the human intestines is associated with obesity, as obese patients had lower F/B ratios than normal individuals [42]. Li et al. (2018) found that a high-fat diet (HFD) can reduce the abundance of S24-7 and Lactobacillus in the intestines of mice [69]. Serino et al. (2012) reported that treatment with prebiotic gluco-oligosaccharide (GOS) can effectively restore the abundance of S24-7 and Lactobacillus after their reduction due to the HFD [70]. In addition, supplementation with RSV can alleviate the dysregulation of intestinal microbiota caused by an HFD and increase the abundance of Lactobacillus and Bifidobacterium in the intestines [71].

Previous studies about BPA exposure in the peri-pregnancy period showed significant effects on the offspring [28,29,72]; however, little research was performed to investigate the effects of the intestinal microbiota on the offspring. According to Xu et al. (2019), non-obese diabetic (NOD) mice (both female and male) that were born with exposure to BPA (300 μg/kg BW) during the peri-pregnancy period did not show significant changes in the Firmicutes/Bacteroidetes (F/B) ratio [73]. Our study also showed a similar finding (see Figure 3A). Although the F/B ratios in the BPA and CN groups showed no significant differences in our study, metabolic abnormalities in female offspring induced by BPA exposure during the peri-pregnancy period were still present (also see [28,29,72]). In the present study, RBE significantly changed the intestinal microflora of the offspring (the F/B ratio decreased) (see Figure 3A). Based on this important finding, we speculate that BPA affected the slow body-fat metabolism of the female offspring and caused obesity, and it could also be possible that the intestinal flora of the female offspring adjusted to return the lipid metabolism to normal because of the RBE. The related physiological metabolic pathways need to be further clarified in the future.

RBE can be catabolized into RSV and butyrate [40]. However, the outcomes of RSV ingestion in pregnant women may differ from those in non-pregnant women. In particular, the intestinal microbiota in the offspring are mainly derived from the maternal microbiota, but the composition of the intestinal microbiota in infants is directly affected by childbirth delivery methods, postnatal diets, and the postnatal environment [74]. Huang et al. [75] showed that RSV supplementation could not alleviate the reduction in the abundance of Lactobacillus in offspring caused by prenatal and postnatal HFDs. Zha et al. (2020) revealed that the offspring of normal mice treated with 40 mg/kg/day of RSV during pregnancy displayed a decreasing trend in the abundance of Lactobacillus in their intestinal tracts [76]. The results of these previous studies were consistent with those of this study (Figure 3B,C), i.e., the results of the operational taxonomic units (OTUs) showed that RBE intake can increase the abundance of S24-7 and decrease the abundance of Lactobacillus. Animal studies on RBE have rarely been reported, and no perinatological studies demonstrating the effects of perinatal supplementation of RBE have been published. Therefore, it is not easy to explore the effects of RBE or its metabolites on obesity, physiology, biochemistry, and intestinal microbiota in female offspring rats without sufficient references.

The results of this study (Figure 3D) revealed that BPA exposure increased the abundance of Prevotella, *Clostridium perfringens*, and *Clostridium ruminantiums*. Treatment with RBE decreased the abundance of Lactobacillus and Prevotella and reduced the abundance of *Clostridium perfringens* and *Clostridium ruminantiums* to normal levels. In addition, the female offspring rats in the B+R30 group showed an increased abundance of Ruminococcus and Adlercreutzia. Chronic inflammation and arthritis are associated with Prevotella, which induces inflammation by stimulating the production of IL-8, IL-6, and CCL20 through colonic epithelial cells [77,78]. *Clostridium perfringens* is believed to be associated with neuromyelitis optica (NMO) and autism. In the intestines, *Clostridium perfringens* can produce enterotoxins that damage the intestinal mucosa and result in malnutrition [79,80]. Therefore, based on our results and others’ studies, we speculated that perinatal exposure to BPA could lead to the dysregulation of intestinal microbiota in female offspring rats, thereby increasing the risk for metabolic abnormalities and intestinal inflammation in offspring.

RBE supplementation can effectively attenuate the BPA-induced dysregulation of intestinal microbiota. In addition, it was found in the correlation analysis performed to examine plasma biochemical markers and the selected microbiota (Figure 3E) that the abundance of Lactobacillus had a positive correlation with TG concentration and LW. Although most Lactobacillus species exhibit an anti-obesity effect, a small proportion of Lactobacillus species may still trigger the onset of obesity and metabolic abnormalities in hosts [81]. In contrast, the abundance of Adlercreutzia was negatively correlated with TG concentration and LW. An increase in the abundance of *Clostridium perfringens* and *Clostridium ruminantiums* may increase the BW and LW of the hosts. Jiao et al. (2019) reported that blueberry polyphenol extract (PPE) exhibits a positive effect on HFD-induced obesity in C57BL/6J mice [82]. Mice fed with an HFD showed weight gain and an increased weight of their adipose tissues, as well as increased lipid metabolism disorders. In contrast, PPE suppressed weight gain and reduced the lipid metabolism to the normal level. Further analysis revealed that PPE altered the composition of the intestinal microbiota in C57BL/6J mice and regulated the abundance of certain bacteria (e.g., Adlercreutzia). Together, these results show that the HFD reduced the abundance of Adlercreutzia, whereas PPE significantly reduced the abundance of Adlercreutzia [82].

It is interesting to note that, in the SCFA analysis, the female offspring in the R30 group (supplemented with RBE alone) showed significantly reduced obesity and adipogenesis but significantly increased faecal levels of acetate. Overby and Ferguson (2021) reported that SCFAs derived from intestinal microbiota can regulate obesity and hypertension by promoting the development of microbiota in hosts [83]. In addition, the R30 group showed a significantly higher faecal concentration of acetate than that of the CN group (Table 2). Mojsak et al. (2020) reported an increase in the faecal concentration of acetate in obese and diabetic patients [84]. Perry et al. (2016) demonstrated that acetate acts on parasympathetic activity to increase food intake and support glucose-stimulated insulin secretion in a rodent model [85]. Thus, we speculate that the acetate content is related to the weight gain caused by R30 supplementation in female offspring rats. In summary, RBE certainly has potential effects in the treatment or regulation of abnormal metabolism in the offspring of mothers exposed to BPA. Continuous and in-depth research is required for a better understanding of the physiological metabolic mechanisms.

## 4. Materials and Methods

### 4.1. Synthesis of Resveratrol Butyrate Ester

Resveratrol butyrate ester (RBE) was synthesised according to the method described by Tain et al. [37,38]. First, resveratrol and butyrate were mixed with tetrahydrofuran, followed by the addition of N-ethyl-N′-(3-dimethylaminopropyl) carbodiimide (EDC) and 4-dimethylaminopyridine (DMAP). Then, the resulting mixture was subjected to esterification in the dark at room temperature for 48 h. Subsequently, a large amount of distilled water was added to the reaction mixture, followed by filtration. The resulting precipitate was rinsed with alcohol to obtain RBE, which was subsequently freeze-dried and stored at −20 °C.

### 4.2. Experimental Animals

The design of this experiment referred to a series of DOHaD trials and proceeded with the principles of the 3Rs for animal experimentation from Tain et al. [23,24,26,86]. Each group of two pregnant female rats will give birth to an average of 20 offspring [87]. Fifteen-week-old pregnant Sprague Dawley (SD) rats (purchased from BioLASCO Taiwan Co., Ltd., Taipei City, Taiwan) were used to construct the experimental models in compliance with the “Guide for the Care and Use of Laboratory Animals” of the National Institutes of Health. This study was approved by the Laboratory Animal Committee of Kaohsiung University of Science and Technology (Approval No: 0108-AAAP-010). All animal experimentations were carried out in animal facilities accredited by the Association for Assessment and Accreditation of Laboratory Animal Care International (AAALAC). The rats were maintained with 12-h light/dark cycles at 25 ± 1 °C. The 15-week-old pregnant SD rats were randomly divided into five groups: the control group (CN), R30 group (administered with 30 mg/kg/day of RBE dissolved in 0.2 mL corn oil), BPA group (administered with 50 μg/kg/day of BPA dissolved in 0.2 mL corn oil), BPA+R10 group (administered with 50 μg/kg/day and 10 mg/kg/day of BPA and RBE, respectively), and BPA+R30 group (administered with 50 μg/kg/day and 30 mg/kg/day of BPA and RBE, respectively). The dose of BPA used here was based on a previous study [72,88]. RBE is a novel synthesis compound, the dose designed in this study referenced the dose used in RSV related studies [72,89,90]. Starting from the sixth day after pregnancy, the rats were subjected to gavage feeding with BPA and/or RBE for 36 consecutive days (including the first 21 days of lactation). Each group of two pregnant female rats gave birth to 14 (CN), 16 (R30), 13 (BPA), 16 (B+R10), 17 (B+R30) offspring, respectively. Subsequently, their female offspring rats (*n* = 4–8 per group) were fed with a regular diet, which comprised 52% carbohydrates, 23.5% proteins, 4.5% fats, 10% ash, and 8% fibres (Fwusow Taiwan Co., Ltd., Taichung, Taiwan) [72]. The female offspring rats were euthanised at the age of 50 days to harvest their blood samples in tubes containing heparin, abdominal fat, and abdominal skin muscle tissues, as well as intestinal faeces, which were then stored at −80 °C for subsequent analyses. Only female offspring were selected from each litter for subsequent experiments to avoid experimental errors attributed to gender differences. The abdominal white adipose tissues (WATs) were harvested and weighed on an electronic precision balance prior to storage in 10% formalin at −80 °C for subsequent experiments.

### 4.3. Plasma Biochemical and Hormonal Analysis

Blood samples harvested from euthanised female offspring rats were centrifuged at 1500× *g* and 4 °C for 10 min, and the resulting supernatant (plasma) was collected for subsequent experiments. In this study, the concentrations of triglycerides (TG), total cholesterol (TC), high-density lipoprotein (HDL), and low-density lipoprotein (LDL) were determined using the Free Fatty Acid Quantification Assay Kit (Abcam, Cambridge, UK) [91], the Cholesterol/Cholesteryl Ester Quantitation Assay Kit (Abcam, Cambridge, UK) [92], and the HDL and LDL/VLDL Cholesterol Assay Kit (Abcam, Cambridge, UK) [93], while the plasma concentration of leptin was measured using the Leptin Rat ELISA Kit (Thermo Fisher Scientific, Waltham, MA, USA) [94] according to the manufacturer’s instructions.

### 4.4. Histopathology

Adipose and subcutaneous tissues harvested from each female offspring rat were immersed in 10% formalin and then dehydrated with a continuous gradient of alcohol. After that, both samples were rinsed with xylene, embedded in paraffin, and divided into sections of approximately 5 μm. The paraffin-embedded tissues were then rinsed with xylene and absolute alcohol, followed by rehydration with a continuous gradient of alcohol. Subsequently, each section was stained with Mayer’s haematoxylin solution and counterstained with eosin. After dehydration with alcohol and rinsing with xylene, each section was scanned with a panoramic slide scanner (MoticEasyScan), and the resulting images were analysed using Motic DS Assistant (4K).

### 4.5. Metagenomics Analysis

Faecal DNA was extracted with the QIAmp Fast DNA Stool Mini Kit (Qiagen, Hilden, Germany) according to the manufacturer’s instructions. The DNA concentration was assessed by a NanoDrop 2000 (O.D. 260/280: 1.7–2.2, Conc. > 50 ng/µL; Thermo Scientific, Waltham, MA, USA) with 10× dilution (final conc.: 4–6 ng/µL) and with an elution buffer. The library was constructed using the standard V3–V4 region of the 16S ribosomal RNA gene. PCR was performed using the KAPA HiFi hotstart readymix (Roche, Indianapolis, IN, USA) and was purified with AMPure XP magnetic beads (Beckman Coulter, Brea, CA, USA). The quality of the amplified PCR product was assessed with a Fragment Analyzer (Advanced Analytical, Ankeny, IA, USA) and was quantified by a Qubit 3.0 Fluorometer. Then, the amplicons were sequenced on a MiSeq platform (Illumina, San Diego, CA, USA) with paired-end reads (2 × 300 nt) and at least 100,000 reads of every sample.

The raw paired-end reads were trimmed, and those that passed the quality filters were clustered into operational taxonomic units (OTUs) at ≥97% similarity with the GreenGene Database (v13.8). The taxonomic (relative abundance, heatmap), alpha diversity (Shannon index, Venn diagram), and beta diversity (PCoA, phylogenetic curve) characteristics of the OTUs were analysed with Basespace (Illumina, San Diego, CA, USA), the CLC genomics workbench (Qiagen, Hilden, Germany), and GraphPad Prism 8 (GraphPad Software, San Diego, CA, USA). The abundance of bacteria (linear discriminant analysis effect size, LEfSe) and associated functions (Phylogenetic Investigation of Communities by Reconstruction of Unobserved States, PICRUSt) were assessed on the Galaxy/Hutlab website (http://huttenhower.sph.harvard.edu/galaxy/) (accesed data on 29 June 2021). A *p*-value of less than 0.05 was considered statistically significant.

### 4.6. Quantification of Faecal SCFAs

To quantify the faecal and colonic content of SCFAs, fresh intestinal contents were freeze-dried before weighing, and then the SCFAs were quantified as reported in the previous study [95]. After that, the mixture was centrifuged at 15,000× *g* and 4 °C for 10 min to obtain the supernatant for a subsequent GC/MS analysis with a DB-FFAP capillary column (30 m × 0.25 mm × 0.25 μm). Injection was performed at 240 °C; the injection volume was 1 μL with a split ratio of 5:1. The identities of compounds were determined by matching their mass spectra with GC/MS databases. Each peak identified via MS spectral matching exhibited a unique retention time (RT) and matching rate in the qualitative analysis (QUAL).

### 4.7. Statistical Analyses

A statistical analysis was conducted with a one-way analysis of variance (ANOVA) and Duncan’s test. Significant differences were set at *p* < 0.05. All statistical analyses were performed using the SPSS (version 12.0, St. Armonk, NY, USA) software.

## 5. Conclusions

In this study, rats were exposed to BPA during the perinatal period to observe the effects of RBE supplementation on obesity-related indicators and intestinal microbiota in their female offspring to better understand the potential application of RBE. The results demonstrated that RBE supplementation can alleviate BPA-induced weight gain and body fat accumulation, optimise the concentration of blood-lipid-related markers, significantly reduce the Firmicutes/Bacteroidetes (F/B) ratio and the abundance of Lactobacillus, and increase the abundance of S24-7. In addition, RBE supplementation can also regulate the intestinal concentration of acetate in female offspring rats. In summary, RBE can suppress BPA-induced obesity in female offspring rats and exhibits excellent modulatory activity in intestinal microbiota, with potential applications in perinatological research. In the future, we will clarify the metabolism of RBE in the digestive systems of animals and its haemodynamics after absorption to further understand the potential of RBE in future applications.

## Figures and Tables

**Table 1:** Body weight, lipid weight, and plasma biochemical parameters of female offspring rats.

Groups	CN	R30	BPA	B+R10	B+R30
Body weight (g)	171.3 ± 2.7 ^a^	189.9 ± 8.2 ^b^	198.1 ± 9.8 ^b^	184.4 ± 6.3 ^ab^	172.9 ± 2.0 ^a^
Lipid weight (g) *	2.21 ± 0.34 ^a^	1.45 ± 0.14 ^a^	3.91 ± 0.88 ^b^	2.17 ± 0.43 ^a^	1.32 ± 0.42 ^a^
Relative lipid weight (%) ^#^	1.29 ± 0.25 ^a^	0.81 ± 0.04 ^b^	2.20 ± 0.30 ^c^	1.25 ± 0.18 ^a^	0.91 ± 0.26 ^a^
TG (μg/mL)	79.5 ± 10.6 ^a^	77.0 ± 10.7 ^a^	132.0 ± 2.0 ^b^	105.0 ± 5.0 ^c^	68.3 ± 5.5 ^a^
TC (μg/mL)	1095.2 ± 59.9 ^a^	1167.2 ± 33.3 ^ab^	1260.5 ± 38.7 ^c^	1281.4 ± 17.2 ^c^	1209.2 ± 9.2 ^bc^
HDL (μg/mL)	143.5 ± 15.8 ^ab^	144.1 ± 12.5 ^ab^	73.7 ± 2.6 ^c^	164.2 ± 5.3 ^a^	134.2 ± 23.3 ^b^
LDL (μg/mL)	667.1 ± 26.7 ^a^	498.3 ± 5.3 ^b^	870.2 ± 40.3 ^c^	536.8 ± 7.9 ^d^	482.8 ± 22.6 ^a^
Leptin (pg/mg)	350.9 ± 31.6 ^a^	419.4 ± 6.0 ^b^	437.6 ± 17.9 ^b^	344.2 ± 12.3 ^a^	355.5 ± 11.2 ^a^

^a–^^d^ Values with different letters are specific to each group and show significant differences (*p* < 0.05). CN = control group; R30 = group administered with 30 mg/kg/day of RBE; BPA = group administered 50 μg/kg/day of bisphenol A; BPA+R10 = group administered with 10 mg/kg/day of RBE and bisphenol A; BPA+R30 = group administered with 30 mg/kg/day of RBE and bisphenol A; * abdominal LW; ^#^ Relative lipid weight (%): (abdominal lipid weight/BW) * 100%. Data are expressed as means ± SD (*n* = 4–8).

**Table 2:** SCFA concentrations in the faeces of female offspring rats.

SCFAs	CN	R30	BPA	B+R10	B+R30
(μmol/g Faeces)					
Acetic acid	21.01 ± 2.03 ^a^	28.51 ± 3.54 ^c^	31.67 ± 2.93 ^bc^	23.65 ± 2.64 ^ab^	17.75 ± 1.15 ^a^
Propanoic acid	3.50 ± 0.30 ^ab^	4.30 ± 0.58 ^ab^	3.62 ± 0.21 ^ab^	3.71 ± 0.64 ^a^	2.60 ± 0.25 ^b^
Butanoic acid	2.16 ± 0.61 ^a^	2.30 ± 1.26 ^a^	1.88 ± 0.96 ^a^	2.34 ± 0.29 ^a^	2.80 ± 1.03 ^a^

^a–^^c^ Values with different letters are specific to each group and show significant differences (*p* < 0.05). CN = control group; R30 = group administered with 30 mg/kg/day of RBE; BPA = group administered with 50 μg/kg/day of bisphenol A; BPA+R10 = group administered with 10 mg/kg/day of RBE and bisphenol A; BPA+R30 = group administered with 30 mg/kg/day of RBE and bisphenol A. Data are expressed as means ± SD (*n* = 4–8).

## Data Availability

Not applicable.

## Sample Availability

Samples of the compounds are available from the authors Chih-Yao Hou.
